# Fabrication of nanocrystal forms of ᴅ-cycloserine and their application for transdermal and enteric drug delivery systems

**DOI:** 10.3762/bjnano.15.42

**Published:** 2024-04-25

**Authors:** Hsuan-Ang Tsai, Tsai-Miao Shih, Theodore Tsai, Jhe-Wei Hu, Yi-An Lai, Jui-Fu Hsiao, Guochuan Emil Tsai

**Affiliations:** 1 Department of Research and Development, SyneuRx International (Taiwan) Corp., 20F-8, No. 99, Sec. 1, Xintai 5th Rd., Xizhi District, New Taipei City 221, Taiwan; 2 Department of Psychiatry and Biobehavioral Sciences, UCLA School of Medicine, 10833 Le Conte Ave, Los Angeles, CA 90095, USA

**Keywords:** ᴅ-cycloserine, drug delivery system, enteric capsules, *N*-methyl-ᴅ-aspartate, nanocrystals, NMDA receptor agonist, transdermal reservoir

## Abstract

ᴅ-cycloserine (DCS), an FDA-approved medicine for the treatment of tuberculosis, is also a partial agonist at the glycine recognition site of *N*-methyl-ᴅ-aspartate (NMDA) receptor and has shown significant treatment efficacy for central nervous system (CNS) disorders including depression, schizophrenia, Alzheimer’s disease, and post-traumatic stress disorder. The physicochemical properties of DCS, however, limit the options of formulation and medicinal applications of DCS, and warrants further investigation for the development of CNS therapeutics. Nanocrystals play an important role in pharmaceutic design and development. The properties of nanocrystals are remarkably different from their bulk material counterpart, attributed to the large surface-area-to-volume ratio which can improve the bioavailability. In this study, for the first time, DCS, a highly water-soluble compound, has formed nanocrystals and this was confirmed by scanning electronic microscopy and X-ray powder diffraction. Furthermore, DCS nanocrystals were applied to several formulations to test their stability and then to the in vitro Franz diffusion test with reservoir patch formulation as well as in vivo pharmacokinetics study with enteric capsules. We tested these formulations regarding their nanocrystal physical properties, size effect, and dissolution rate, respectively. We found that DCS nanocrystals showed good performance in the Franz diffusion test and rodent pharmacokinetic studies due to the nanoparticle size and faster dissolution as compared with the commercial DCS powder. These DCS nanocrystal formulations could offer a new approach for the development of an advanced drug delivery system for the treatment of CNS disorders.

## Introduction

Tuberculosis (TB) is a prevalent respiratory disease caused by *Mycobacterium tuberculosis*. According to the Global 2020 Tuberculosis Report by the World Health Organization (WHO), a total of 1.4 million people died of TB [1]. ᴅ-cycloserine (DCS; ᴅ-4-amino-3-isoxazolidone), a cyclic analog of ᴅ-alanine, has been one of the remedies to treat TB since late 1950s, and is on the WHO list of essential medicines against TB [2]. The utilization of Seromycin (250 mg of DCS) in such infections is taken into consideration when conventional therapies fail. Moreover, the medicinal application of DCS is not limited to anti-infection treatments. The efficacy of DCS on central nervous system (CNS) disorders has been studied over the last three decades, primarily due to its centrally active partial agonism of *N*-methyl-ᴅ-aspartate (NMDA) receptor [3–12]. Several researches indicated that DCS is a potential drug candidate to treat CNS disorders such as depression, schizophrenia, Alzheimer's disease, and post-traumatic stress disorder [13–16]. In general, solid dosage forms for oral administration are preferable for therapies against CNS disorders. Nevertheless, challenges persist with oral solid dosage forms, including issues of bioavailability and clinical efficacy [17]. On the other hand, only a few research articles reported DCS formulations for parenteral administration [18–19].

Nanocarriers offer great advantages to many technological fields. For example, polytetrafluoroethylene (PTFE) with silicon carbide nanocrystals can be applied as a photostabilizer or as a UV light absorber [20]. In addition, silver (Ag) nanoparticles were synthesized from cotton fabrics and exhibited strong inhibition activity against some bacteria [21]. Recently, pure active pharmaceutical ingredient (API) composed of nanocrystals was investigated, as opposed to drug nanocarrier platforms [22]. Nanocrystals can improve many physicochemical properties of drugs such as solubility, size effect, dissolution rate, and adhesiveness to surface membranes [23].

The limitations of conventional medication delivery can be overcome by advanced drug delivery methodologies, such as transdermal drug delivery (TDD) and enteric formulation. In this study, DCS nanocrystals were fabricated and investigated for novel drug delivery systems. Transdermal drug delivery is an administration route wherein the API is delivered across the skin for systemic distribution. The categories of TDD systems include reservoir, matrix, and microneedle systems [24–26]. In addition, enteric-coated solid dosage forms for oral administration have shown significant improvement in providing better absorption and targeted delivery [27–28].

In this study, due to high water solubility of DCS (Log *P* = −1.72) and difficulty to dissolving DCS in organic solvents to fabricate the matrix patch formulation, only reservoir patch formulations of DCS in well-suspended (DCS nanocrystals) or emulsified (DCS water solution) forms were feasible to evaluate transdermal drug delivery. Coating drugs with pharmaceutically biocompatible enteric polymers can provide gastric protection against gastric-irritating compounds and/or stomach acidity, leading to improved drug release performance. We aimed to fabricate DCS nanocrystals and study their physicochemical and biological properties. The DCS already has great water solubility (Log *P* = −1.72), so DCS nanocrystals will not have improved water solubility in comparison with that of commercial DCS. Accordingly, we focused on the other two important characteristics of nanocrystals: size effect and dissolution rate, and then investigated the pharmaceutical applications of DCS nanocrystals through in vitro transdermal delivery and in vivo pharmacokinetic (PK) studies.

## Materials and Methods

### Preparation of DCS nanocrystals

A saturated DCS aqueous solution was prepared by dissolving 100 mg of DCS powder (Stride Pharma Science Ltd.) in 1 mL of deionized water. Then, 10 mL of the saturated DCS solution was slowly added into 20 mL of *tert*-butanol (Merck-Millipore) and mixed with an ultrasonic cleaner (Creworks Co., Ltd.) at 40 kHz for 1 min. The mixed solution was filtered by suction filtration using a 0.2 μm filter membrane (Merck KGaA). The filtered solution was centrifuged at 10,000 rpm (Kubota Co., Ltd.) for 10 min. The collection was vacuum-dried to obtain DCS nanocrystals.

### Characterization of DCS nanocrystals

The DCS nanocrystals were analyzed via scanning electronic microscopy (SEM, JEOL Ltd.) and X-ray powder diffraction (XRPD, Bruker AXS GmbH). For SEM, commercial DCS was spreaded onto a double-coated carbon conductive tape and DCS nanocrystals were suspended in ethanol (Merck KGaA) at 10 mg/mL, put on the double-coated carbon conductive tape, and coated with platinum using an auto-fine coater for better electrical conduction. For the XRPD analysis, commercial DCS powder and DCS nanocrystals were scanned in continuous mode from 5–40° (2θ) with a step size of 0.02° on a spinning stage at 40 kV and 40 mA with Cu Kα radiation. The incident beam path was equipped with a 0.2 mm divergence slit and a 0.02 mm air scattering screen. The diffracted beam was equipped with Ni-filter. The detection was accomplished with a LYNXEYE detector (Bruker AXS GmbH), and the XRPD patterns were obtained on a D8 ADVANCE (Bruker AXS GmbH).

### Preparation of different hydrophobic formulations mixed with DCS aqueous solution for the reservoir transdermal delivery

The DCS aqueous solution was mixed with different hydrophobic solutions to obtain emulsion solutions, and loaded into the donor compartment of the Franz diffusion cell for 24 h. The release of DCS was detected in the receptor compartment. The summary of DCS formulations and release is shown in Table 1 and described as:

**Formulation Test 1:** 20 mg of commercial DCS powder was dissolved in 1mL of deionized water.

**Formulation Test 2:** 20 mg of commercial DCS powder was dissolved in 0.45 mL of deionized water and then mixed with 0.2 mL of PEI 600 (Sigma-Aldrich) and 0.3 mL of corn oil. A volume of 0.05 mL of 0.1 N NaOH (Sigma-Aldrich) was used to adjust the pH value (pH 7) of this formulation.

**Formulation Test 3:** 20 mg of commercial DCS powder was dissolved in 0.45 mL of deionized water with 0.2 mL of PEG 400 and then mixed with 0.3 mL of corn oil (Sigma-Aldrich). A volume of 0.05 mL of 0.1 N NaOH was used to adjust the pH value (pH 7) of this formulation.

**Table 1:** Preliminary test of DCS solution in the reservoir transdermal system. Each experiment was tested in triplicate, and the data of DCS releasing rate were presented as mean ± standard deviation.

Formulation test	DI water	PEI600	PEG400	Corn oil	NaOH 0.1 N	DCS releasing rate after 24 h

1	1.00 mL	0.00 mL	0.00 mL	0.00 mL	0.00 mL	0.00%
2	0.45 mL	0.20 mL	0.00 mL	0.50 mL	0.05 mL	0.24 ± 0.01%
3	0.45 mL	0.00 mL	0.20 mL	0.30 mL	0.05 mL	0.36 ± 0.03%

### The pH stability test of DCS

We prepared two solutions for pH stability test to simulate the gastrointestinal environment: 0.1 M of an HCl solution (pH 1.2 at the stomach) (Sigma-Aldrich) and 0.1 M of phosphate buffer (pH 7.4 at the terminal ileum) (Sigma-Aldrich) [26]. Approximately 5 mg of DCS was dissolved in 5 mL of the aforementioned solution at physiological temperature of 37 °C for 1 h. The final amount of DCS was analyzed and calculated by liquid chromatography with tandem mass spectrometry (LC-MS/MS). The remaining percentages (RP%) of DCS were calculated by the following formula:




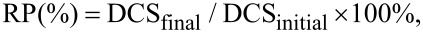




where DCS_final_ and DCS_initial_ represent the final and initial amounts of DCS, respectively.

### Preparation of DCS nanocrystals and commercial DCS powder loaded with different hydrophobic formulations for the reservoir transdermal delivery

The DCS nanocrystals as well as commercial DCS powder (Stride Pharma Science Ltd.) were formulated for the study of reservoir transdermal delivery. The preparation of the transdermal formulation was described below and summarized in Table 2.

**Patch formulation A:** 10 mg of povidone was added to 0.05 mL of NaOH solution. The povidone solution was then mixed with 0.5 mL of dipropylene glycerol (Sigma-Aldrich), 0.6 mL of PEG 400 (Sigma-Aldrich), and 0.2 mL of corn oil (Sigma-Aldrich) to form an emulsified solution by vortexing and sonication. An amount of 15 mg of DCS nanocrystals was added to this emulsion by stirring. After the DCS nanocrystals were well suspended, the final emulsified solution was added to the donor part of the Franz diffusion cell system.

**Patch formulation B:** 5 mg of mannitol and 10 mg of povidone were added to 0.05 mL of NaOH solution. This mannitol/povidone solution was then mixed with 0.2 mL of dipropylene glycerol, 0.8 mL of PEG 400, and 0.3 mL of corn oil to form an emulsion by vortexing and sonication. Then, 30 mg of DCS nanocrystals was added to this formulation by stirring. When the DCS nanocrystals were well suspended, the final product was added to the donor part of the Franz diffusion cell system.

**Patch formulation C:** 5 mg of mannitol and 10 mg of povidone were added to 0.05 mL of NaOH solution. An amount of 0.5 g of PEG 1000 (Sigma-Aldrich) was dissolved in 0.5 mL of dipropylene glycerol. The PEG 1000 solution, mannitol (Sigma-Aldrich)/povidone (Sigma-Aldrich) solution, and 0.45 mL of corn oil were mixed together to form an emulsion by vortexing and sonication. Next, 30 mg of DCS nanocrystals was added to 1.00 mL of the emulsion by stirring. When the DCS nanocrystals were well suspended, the final formulation was added to the donor part of the Franz diffusion cell system.

**Patch formulation D:** 5 mg of mannitol and 10 mg of povidone were added to 0.05 mL of NaOH solution. This mannitol/povidone solution was then mixed with 0.2 mL of dipropylene glycerol, 0.8 mL of PEG 400, and 0.3 mL of corn oil to form an emulsion by vortexing and sonication. Then, 30 mg of commercial DCS powder was added to the 1.35 mL emulsion by stirring. When the commercial DCS powder was well-suspended in the emulsion, the final formulation was added to the donor part of the Franz diffusion cell system.

**Patch formulation E:** 10 mg of povidone was added to 0.05 mL of NaOH solution. The povidone solution was then added to 0.5 mL of dipropylene glycerol, 0.6 mL of PEG 400, and 0.2 mL of corn oil to form an emulsion solution by vortexing and sonication. Then, 15 mg of commercial DCS powder was added to the emulsion by stirring. When the commercial DCS powder was well mixed and suspended in the formulation, the final formulation was added to the donor part of the Franz diffusion cell system.

**Table 2:** The formulations for reservoir transdermal system of DCS and the results of the releasing study in vitro. Each formulation was tested in triplicate, and the data of DCS release and *T*_1/2_ were presented as mean ± standard deviation.

Patch Formulation	DPG^a^ (mL)	NaOH (mole)	PEG (mL)	Corn oil (mL)	Mannitol (mg)	Povidone (mg)	DCS (mg/mL)	Maximum DCS Release (mg)	*T*_1/2_ (h)

A	0.50	0.75	0.60^b^	0.20	0.00	10.00	11.11^d^	6.42 ± 1.21	40 ± 10
B	0.20	0.10	0.80^b^	0.30	5.00	10.00	22.22^d^	10.09 ± 0.32	46 ± 2
C	0.50	0.10	0.50^c^	0.45	5.00	10.00	30.00^d^	11.23 ± 0.38	32 ± 3
D	0.20	0.10	0.80^b^	0.30	5.00	10.00	22.22^e^	0.53 ± 1.62	37 ± 6
E	0.50	0.75	0.60^b^	0.20	0.00	10.00	11.11^e^	0.16 ± 0.20	77 ± 24

^a^DPG: Di-propylene glycerol; ^b^PEG400; ^c^PEG 1000; ^d^DCS nanocrystal; ^e^Commercial DSC.

### Formulation of nanocrystal DCS and commercial DCS powder in enteric capsules

Size 9 empty porcine hard gelatin capsules (Torpac) were used for the following experiments. The modified enteric capsules contained one of the nanocrystal forms or the commercial DCS powder, which were prepared for in vivo PK study. The detailed compositions for the capsule formulations are described next.

**Enteric capsule formulation F:** Kollicoat MAE 30 DP (BASF pharma), a reliable, high-performance film coating based on methacrylic acid-ethyl acrylate copolymer (1:1), is specifically designed for enteric solid dosage coating and is used in this work for DCS enteric administration test. An amount of 5.66 g of Kollicoat MAE 30 DP was mixed with 0.34 g of propylene glycol for the enteric coating material, which was evenly brushed on the surface of the size 9 empty porcine hard gelatin capsules (Torpac), and then air-dried until the coated capsules hardened. After repeating the coating and drying cycles for five times, the opened capsules were ready to use. Approximately 10 mg of the DCS nanocrystals was filled into each capsule.

**Enteric capsule formulation G:** The formulation G was prepared by the same method used in formulation F, except that the DCS nanocrystals were substituted with the commercial DCS powder.

### In vitro transdermal delivery study

A Strat-M membrane (pore size = 450 nm, Merck KGaA) was mounted on a Franz diffusion cell (PermeGear). The receptor compartment contained 8 mL of deionized water. Approximately 1–1.35 mL of the transdermal formulations (Formulation tests 1–3 and A–E) were applied to the membrane over an area of 1 cm^2^ across the donor compartment. The donor cell was exposed to room temperature with 65% humidity and covered with Parafilm to prevent evaporation. The deionized water was continuously stirred with a Teflon-coated magnetic bar at 32 °C. The samples (0.2 mL) were withdrawn from the release medium at 6, 24, 48, 72, 96, 120, and 144 h and were replaced with an equal volume of deionized water to maintain the filtration. The samples were diluted with methanol and then analyzed by LC-MS/MS.

### In vivo pharmacokinetic study of the enteric formulation

The study protocol and the standard operating procedures (SOPs) were reviewed and approved by the Institutional Animal Care and Use Committee (IACUC) of Eurofins. All aspects of this study including housing, experimentation, and animal disposal were performed in general accordance with the Guide for the Care and Use of Laboratory Animals (National Academy Press, Washington, DC, 2011). In addition, Eurofins (Taiwan site) conducted the animal studies. Eight-week-old Sprague Dawley rats were randomly assigned into two groups with three rats in each group. All rats were fasted overnight with free access to water before the administration of the formulations. For the PK study, single doses of DCS nanocrystals or of the commercial DCS powder in the enteric formulation (Enteric Formulation E and F, respectively) were orally administrated in rats. A volume of 300 µL of rat blood was collected at each of the nine timepoints, and a total of 2.7 mL of blood was collected from each rat within 24 h. Blood samples were processed to obtain plasma (more than 150 µL) for subsequent analysis. Plasma samples were analyzed by LC-MS/MS to determine the amount of DCS.

### Liquid chromatography with tandem mass spectrometry for DCS analysis

The stationary phase of liquid chromatography (LC) was a 5 µm C18 column from Thermo Fisher Scientific (15 cm × 4.6 mm). The mobile phase A was comprised of acetonitrile (Merck Millipore) and 0.1% of trifluoroacetic acid (Merck Millipore), and the mobile phase B was comprised of deionized water and 0.1% of trifluoroacetic acid. The gradient of mobile phases was set with a flow rate of 0.5 mL/min. The temperature of the autosampler was set at 4 °C, and the injection volume was 5 µL. The total run time of the LC was 20 min. Ionization and detection of the analyte were performed on a triple quadrupole mass spectrometer (API-2000, AB Sciex Pte. Ltd.), operating in positive ion mode. Quantitation was done using multiple reaction monitoring (MRM) mode to monitor protonated precursor → ion transition product of *m*/*z* 103.1 → 75.0 for DCS of this study. The concentration range of DCS for the calculation curve was from 0 to 0.3 mg/mL. The standard curve was built as *y* = 2E+07*x* + 43665, and the value of *r*^2^ was 0.9986.

### Statistical analysis

Each transdermal formulation in the in vitro release study and in the PK study for enteric capsules was performed in triplicates for statistical analysis. The data were presented as mean ± standard deviation (SD) in the tables. The *p*-value was calculated through Student’s *t*-test to determine the statistical significance.

## Results

### Characterization of the DCS nanocrystals

Previous studies showed that polymorphisms can be induced by changing the solvent, the temperature of the experiment, and the substance concentration [29]. The synthesis of precipitated particles also depends on the concentrations of components, the combination of solvent and antisolvent, and the mixing conditions. During precipitation, different solvents can affect the size, the structure, and the stability of polymorphous particles.

**Figure 1:**
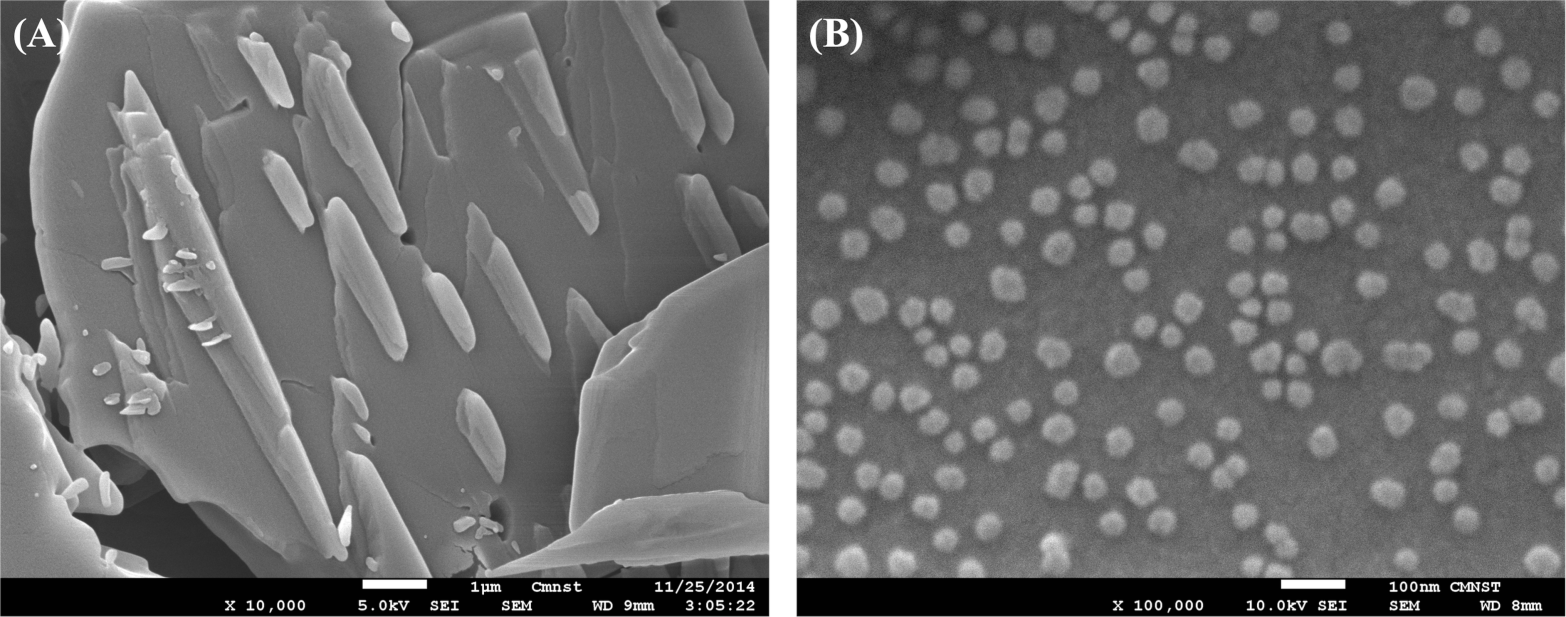
Scanning electron microscopy images of commercial DCS (A) and DCS nanocrystals (B).

To confirm the morphology of DCS nanocrystals, SEM and XRPD analyses were applied for the examination of particle size and structure, respectively. As illustrated in Figure 1 and quantified using ImageJ, the particle size of commercial DCS is in the range of several hundred microns, as depicted in Figure 1A. Conversely, the average particle size of DCS nanocrystals is 47.3 ± 9.5 nm, as evidenced in Figure 1B. This observation indicates a distinct morphology of DCS nanocrystals when compared to that of commercial DCS.

**Figure 2:**
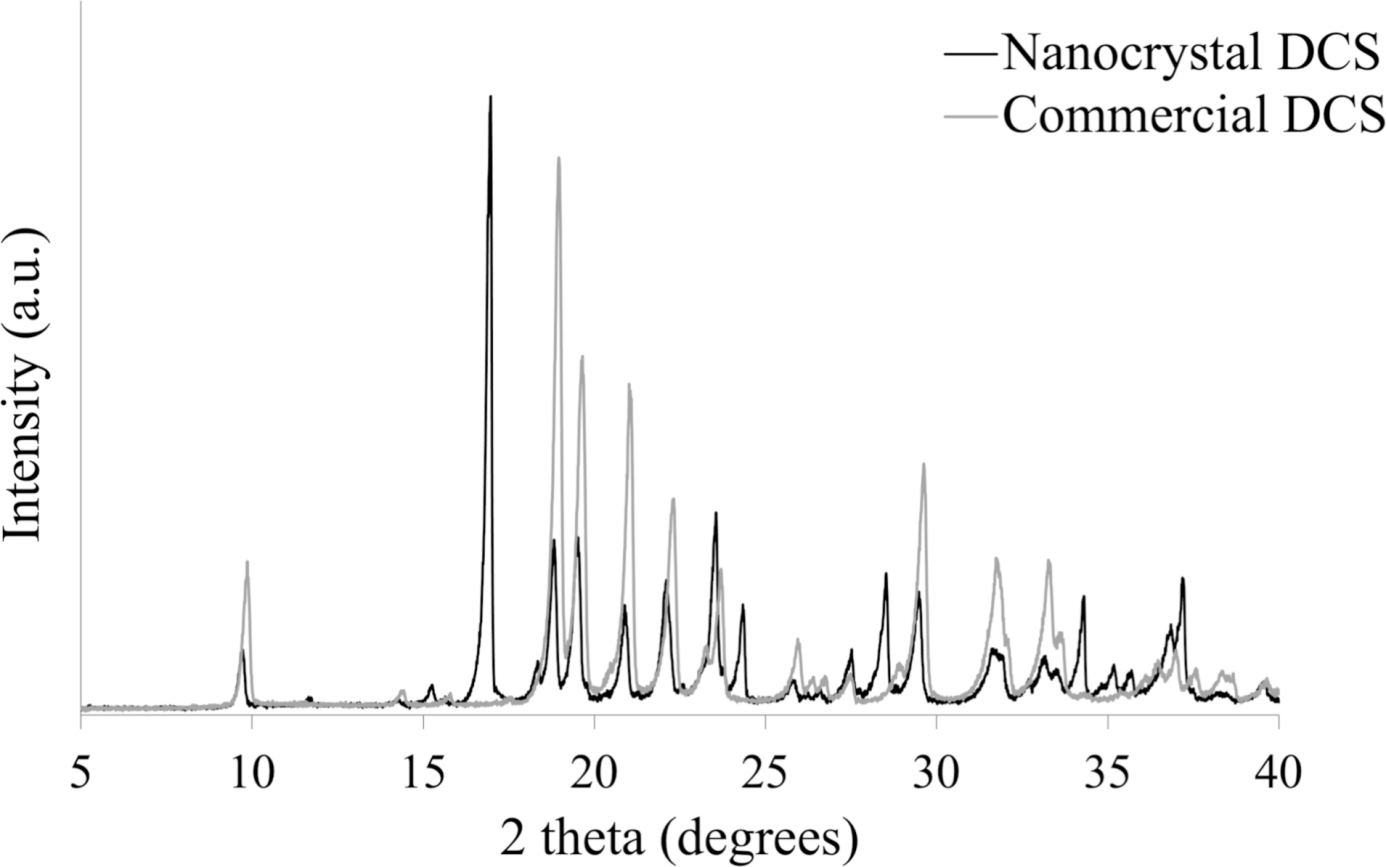
The X-ray powder diffraction spectra of DCD nanocrystal and commercial DCS.

In order to confirm whether the crystalline DCS was a new crystal form, the XRPD patterns of the DCS nanocrystals were compared with those of the commercial DCS powder. As shown in Figure 2, the prominent peaks of the DCS nanocrystals were at 17.0°, 24.3°, 28.5°, and 34.3° (2θ), distinct from those of commercial DCS. These results indicated that the new crystalline DCS was a novel nanocrystal.

The spectra demonstrate that the nanocrystal form of DCS was successfully generated. To exclude crystal polymorphism, the morphology and stability of the DCS nanocrystals used in this study were confirmed by SEM and XRPD before the experiments.

### The excipient compatibility study and the pH effect on DCS stability

The objective of the DCS excipient compatibility test (Table 1) was to determine the best excipient, the optimal pH range, the best storage conditions for the formulation of the DSC aqueous solution, and any potential incompatibilities between the DSC aqueous solution and other excipients.

We found that without adding NaOH solution to the Formulation Test 2 and 3 in Table 1, the color of the DCS solution changed from colorless to dark brown within approximately 10 min.

**Figure 3:**
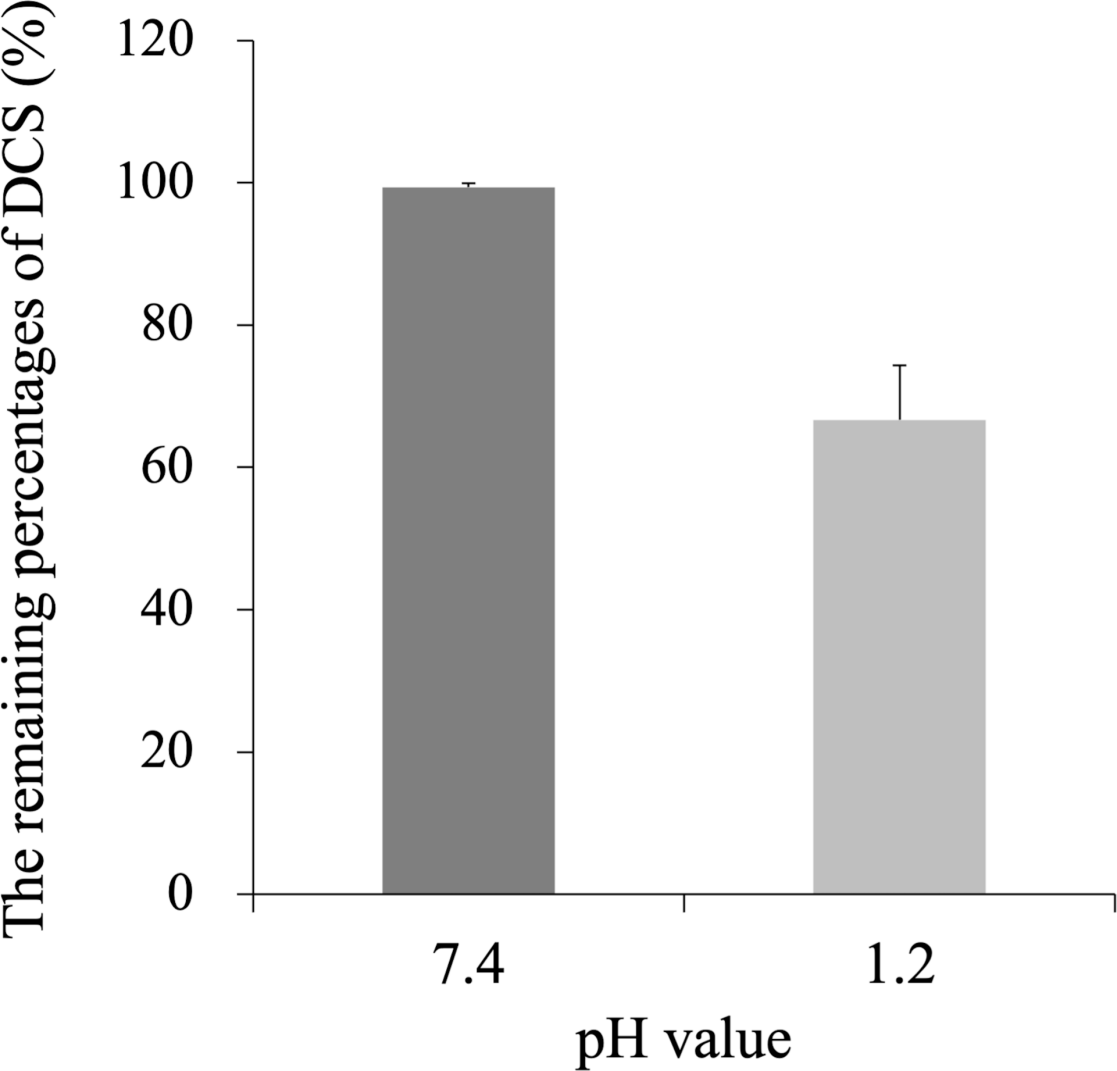
The pH stability test of DCS in neutral (pH 7.4) and acidic (pH 1.2) environments (*p* < 0.01).

As shown in Figure 3, the amount of DCS was remarkably reduced by 40% compared to the initial amount in an acidic environment simulating the stomach (pH 1.2). This finding is consistent with a previous study showing that DCS is sensitive to acidic conditions and can be hydrolyzed and decomposed into hydroxylamine and ᴅ-serine [17].

Thus, controlling the pH of the formulation is essential for obtaining a stable product. Therefore, an enteric formulation was warranted for DCS nanocrystals in order to achieve targeted delivery and improved bioavailability.

### In vitro transdermal delivery of a DCS aqueous solution with hydrophobic formulations

The transdermal formulation of DCS should demonstrate long-term penetration capability and stability in the Franz diffusion cell system. In the reservoir transdermal delivery system, compounds were mixed in a compartment and well suspended in liquid (i.e., the compounds became an emulsion solution or suspension liquid) or gel form [30]. Deionized water was used as a receptor medium for drug penetration tests [31–32]. To study the in vitro release of DCS in different crystal forms, the reservoir transdermal delivery system was used to determine the effect of DCS nanocrystals and of the commercial DCS products. The results of the DCS formulation test were summarized in Table 1. Despite its hydrophilic property (Log *P* = −1.72), DCS could not be released into the receptor compartment after 24 h (Formulation Test 1). Similar results were observed in Formulation Tests 2 and 3. The release of DCS for these two formulations was 0.24% and 0.36% after 24 h, respectively. Furthermore, in the Formulation Tests 2 and 3, two separate layers were observed from the residual solutions in the donor compartment. In summary, both the DCS aqueous solution and mixed emulsion solution were not appropriate formulations for transdermal delivery.

### In vitro transdermal delivery of DCS nanocrystals and commercial DCS powder with different hydrophobic formulations

When we applied DCS nanocrystals and commercial DCS powder to the hydrophobic formulation, we fabricated transdermal delivery systems with a solid-dispersed suspension. The release of DCS in the Franz diffusion cell system was significantly increased compared to the that of the DCS aqueous solution mixed with the hydrophobic formulation. The kinetic findings for both transdermal release of DCS nanocrystals and commercial DCS powder were simulated by the non-linear equation. Table 2 summarizes the simulated maximum release amount and *T*_1/2_ (time when half-maximum DCS is released) for each formulation.

**Figure 4:**
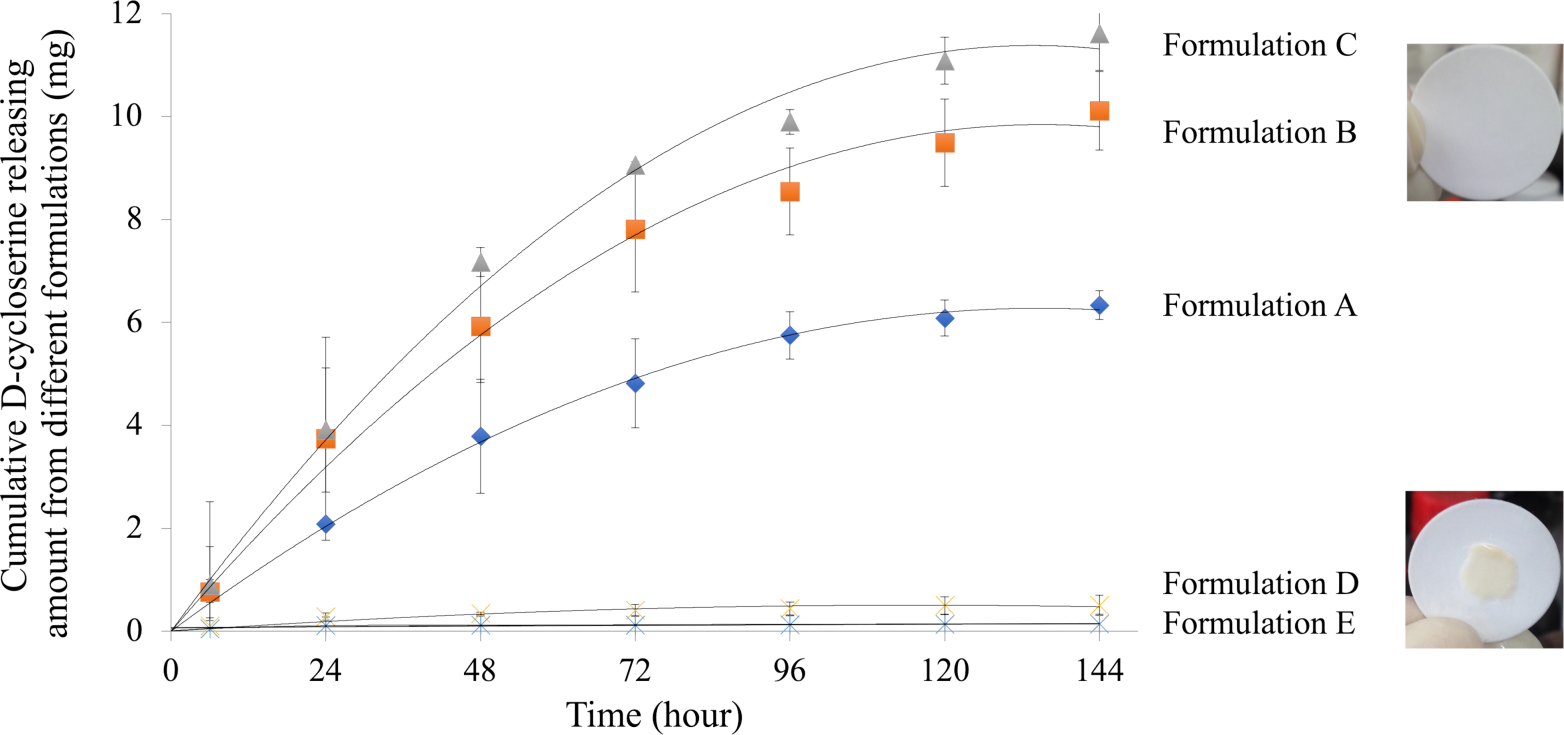
In vitro long-term transdermal release kinetics of DCS nanocrystals and commercial DCS. (A–E are patch formulation compositions loaded with nanocrystals or commercial DCS that have been described in the section "Preparation of DCS nanocrystals" (*n* = 3)).

We first tested the release ability of the commercial DCS powder. The Formulation E exhibited a small amount of DCS penetration when a low concentration of the commercial DCS powder was loaded. Interestingly, by simply increasing the concentration of DCS powder and adding mannitol as an enhancer (Formulation D), the release of DCS significantly increased from 0.16 ± 0.20 mg to 0.53 ± 1.62 mg (Table 2). Similar results were found between Formulations A and B when the DCS nanocrystals were loaded in the transdermal delivery system. These results suggested that passive transport may be one of the driving forces for the penetration of both DCS nanocrystals and commercial DCS powder. In addition, mannitol is an excipient that enhances the absorption of formulations such as oral, injectable, inhaled, and transdermal delivery [33–36]. In our study, mannitol also promoted the penetration of DCS in the reservoir transdermal delivery in vitro. Excellent release was also observed in similar formulations loaded with DCS nanocrystals. The DCS nanocrystals exhibited substantially higher performance in transdermal delivery efficiency compared to that of commercial DCS powder. As shown in Table 2 and Figure 4, in the long-term release experiment, DCS nanocrystals (Formulation B) showed a 19-fold improvement in penetration, with a similar releasing rate (*T*_1/2_), as compared to the commercial DCS powder (Formulation D). Besides, a cake formation of the commercial DCS powder formulation was obvious on the transdermal membrane after six days of experimentation, whereas no cake formation was observed for the DCS nanocrystal formulation. Moreover, the DCS nanocrystal formulation remained colorless throughout the six-day releasing tests. This observation suggested that DCS nanocrystals were stable in these formulations.

A recent study has reported that the gaps between tumor vascular endothelial cells are at low frequency, and that the trans-endothelial pathways are the dominant mechanisms for nanoparticle extravasation in tumors (also called enhanced permeability and retention (EPR) effect) [37]. Since the skin has a denser structure than that of tumor vessels, we speculated that a driving force similar to the EPR effect might be one of the forces driving DCS nanocrystals to penetrate the skin layer.

The findings above implied that DCS nanocrystals had a much better penetration capability through human skin and could be applied for transdermal drug delivery due to their smaller particle size. In addition, we used Quality by design (QbD) to generate parameters that can be applied to commercial products in the future. The QbD is a systematic approach to drug development that ensures that a product is of high quality and consistently meets the desired characteristics throughout its life cycle. The QbD development process in the pharmaceutical field typically consists of three main steps: (1) the definition of a quality target product profile (QTPP); (2) the identification of critical material attributes (CMAs), critical quality attributes (CQAs) and critical process parameters (CPPs); and (3) the initial risk assessment (RA) and RAs after development [38]. In this study, the experimental data were all from laboratory research. After analysis of these preliminary data, only few parameters of QTPP and CQAs can be roughly generated, as listed in Table 3 and Table 4, respectively. Other parameters require more detailed development experiments, and process analysis will be developed in the future.

**Table 3:** Quality target product profile for DCS nanocrystal formulation.

QTTP elements	Target	Justification

Dosage form	Reservoir path	The proposed dosage form
Dosage design	Sustained release	For 6 days continuous release by Franz diffusion cells system
Route of administration	Transdermal	As proposed
Dose strength	Dose	From PK study
Drug product quality attributes	Color and appearance	Meets the requirements
	Drug permeation	
	pH	
	Particle size	

**Table 4:** Critical quality attributes for DCS nanocrystal formulation.

CQA	Specification	Justification

Color and appearance	Colorless and nanocrystal suspension in the solution	Color and appearance are critical attributes for stability and permeation tests. When the color changes, stability fails. When there is a precipitation in the solution, drug permeability reduces.
Drug permeation	Maximum release amount above 10 mg	For six days of continuous drug release by Franz diffusion cells system; using non-linear regression to calculate the maximum amount of drug release.
pH	pH 7	The pH is compatible with the skin (pH 4 to 7)
Particle size distribution	Particle size bellow 200 nm	Particle size is a key factor for DCS delivery. When the particle size is in the nanometer scale, its permeability is significantly enhanced.

### Rodent pharmacokinetic study for different crystal forms of DCS with oral formulation and enteric coating

The findings of rat PK study, as shown in Table 5, revealed that DCS nanocrystals in enteric capsules had distinct PK parameters compared to those of commercial DCS.

**Table 5:** The PK parameters of the nanocrystals and the commercial DCS in size 9 enteric capsule orally administered to rats (*n* = 3, the data were presented as mean ± standard deviation. *p* < 0.05 in *T*_max_ and *C*_max_, and *p* < 0.10 in AUC_Inf_).

Formulation	*T*_max_ (h)	*C*_max_ (ng/mL)	AUC_last_ (h·ng/mL)	AUC_Inf_ (h·ng/mL)

F (nanocrystals)	0.833 ± 0.557	94068 ± 4853	141669 ± 6668	141752 ± 6621
G (commercial)	1.167 ± 0.557	74132 ± 9694	129027 ± 12183	129121 ± 12219

Compared to the PK parameter of commercial DCS powder (*T*_max_ = 1.17 h and *C*_max_ = 74132 ng/mL), the *T*_max_ (0.83 h) and *C*_max_ (94068 ng/mL) of DCS nanocrystals were 29.06% shorter and 26.89% higher, respectively (*p* < 0.05). In addition, the AUC_Inf_ of DCS nanocrystals (141752 h·ng/mL) was 9.78% higher than that of the commercial DCS powder (129121 h·ng/mL) (*p* < 0.10). These results indicated that DCS nanocrystals had better kinetic properties and bioavailability than the commercial DCS powder.

## Discussion

The DCS was labile and prone to decomposition in an acidic environment (Figure 3). Therefore, we developed enteric formulations of both DCS nanocrystals and commercial DCS powder to reduce the acid lability. Our PK results indicated that, compared to the commercial DCS powder, DCS nanocrystals had better bioavailability (as shown by *T*_max_, *C*_max_, and AUC) in the enteric formulation. Shorter *T*_max_, (0.83 h) represented faster dissolution of DCS nanocrystals, making it a more efficient and rapid treatment. In addition, another enteric tablet formulation of DCS also needs to be developed [39].

The skin is the largest organ of the human body and acts as a great protective barrier against the entry of suspicious microbial species and foreign materials. This functional barrier is a result of the highly hydrophobic nature and compact feature of the outermost skin layer with a broad pH range from pH 4.0 to pH 7.0 [40]. This robust protection, however, hinders the transdermal delivery of hydrophilic drugs, such as DCS (Log *P* = −1.72), and impedes the utilization of these medications [41]. Furthermore, transdermal delivery processes mainly contain two steps: first, the drug penetrates the skin layer; second, the drug dissolves and is loaded into the blood reaching systemic circulation. Notably, the first step is the key barrier for an efficient drug delivery. In this study, the application of Franz diffusion cells, a standard procedure of transdermal in vitro testing in the USP-NF<725>, helps us to test the penetration of drugs. Aqueous solution or mixed emulsion solution of DCS were not appropriate formulations for penetrating the Franz diffusion system due to their high hydrophilicity. We found a suitable hydrophobic composition to overcome this barrier. In addition, it has been reported that nanoparticles with a size below 40 nm can successfully penetrate the skin in which the intercellular space of skin stratum corneum is around 10–100 nm [42]. Taken together, we fabricated DCS nanocrystals and immersed them into hydrophobic formulations to form a solid dispersion for transdermal delivery systems. These formulations significantly promoted the penetration of DCS with long-term release, as compared to the formulation with commercial DCS powder in the Franz diffusion system (shown in Figure 4). In the specific formulations (patch formulations A–C), our findings showed that DCS nanocrystals could efficiently penetrate the highly hydrophobic membrane (Strat-M membrane) with a favorable long-term release time profile. In our study, the DCS nanocrystals in the transdermal patch provided a sustained release for six days. Moreover, the DCS nanocrystal formulation, which showed no color change, suggested the stability of DCS nanocrystals under these experimental conditions. This is the first time that DCS nanocrystals were successfully developed and applied in a transdermal system. Stability tests of these formulations under high temperature and high humidity for final formulations can be conducted in the future. In addition, we found that combining passive transport and EPR-like force allowed DCS nanocrystals to overcome their high hydrophilic properties and penetrate the skin layer. These formulations can be applied as a reservoir patch system for long-term transdermal delivery.

In summary, DCS nanocrystals administered by either transdermal or oral routes with enteric coating can be developed as novel and effective formulations for the treatment of human infections and CNS disorders.

## Conclusion

In this study, we found that DCS nanocrystals have better bioavailability than the that of native commercial DCS, which is attributed to specific physicochemical properties of the nanocrystals, including faster dissolution and greater resistance to degradation in enteric capsules. Furthermore, with efficient delivery and long-term stability, the hydrophobic formulation of DCS nanocrystals is a potent formulation in the transdermal delivery system.
